# Efficacy of convalescent plasma therapy in improving survival in non-immunized COVID-19 patients

**DOI:** 10.1186/s12985-025-02778-8

**Published:** 2025-06-24

**Authors:** Tania Portella Costa, Dayanne Mozaner Bordin, Mateus Nóbrega Aoki, Lucas Blanes

**Affiliations:** 1 Epidemiology and Health Surveillance Center, Oswaldo Cruz Foundation, Avenida L3 Norte, Brasília, Brazil; 2 Atomic Medicine Initiative (AMI) & HyMaS Laboratory, School of Mathematical and Physical Sciences, University of Technology Sydney, 15 Broadway, Ultimo, NSW 2007 Australia; 3 Laboratory for Applied Science and Technology in Health, Carlos Chagas Institute, Oswaldo Cruz Foundation (Fiocruz), Professor Algacyr Munhoz Mader 3775 St, Curitiba, Paraná, ZIP 81350-010 Brazil

## Abstract

**Purpose:**

Convalescent plasma (CP), obtained from individuals who have recovered from COVID-19, has been widely explored as a potential therapeutic option, particularly in the absence of vaccines and monoclonal antibody treatments. This study aimed to evaluate the effectiveness of CP therapy in improving survival among non-immunized COVID-19 patients hospitalized in Brazil.

**Methods:**

This retrospective unicentric cohort study was conducted at a private hospital in Campo Largo, Paraná, Brazil, from July 2020 to February 2021. A total of 245 hospitalized COVID-19 patients were included, confirmed by RT-qPCR or antigen testing. Patients were divided into two groups: those receiving CP alongside standard treatment (n=100) and those receiving standard treatment alone (n=145). Survival outcomes were assessed using Kaplan-Meier analysis and Cox regression, while inflammatory responses were evaluated through C-reactive protein (CRP) measurements.

**Results:**

Patients treated with CP had a significantly higher survival rate (91%) compared to the control group (82.8%) (P=0.0363). The survival benefit persisted throughout the follow-up period, with a 2.25-fold lower risk of death in the CP group after adjusting for age (P=0.0480). However, no significant differences in CRP levels were observed between groups at discharge, suggesting that CP's benefits may be mediated through immune modulation rather than direct anti-inflammatory effects.

**Conclusions:**

Our findings indicate that CP therapy significantly improves survival in non-immunized COVID-19 patients, reinforcing its potential role in settings with limited access to advanced treatments. Future studies should explore CP’s mechanisms of action and its integration into broader therapeutic strategies.

## Introduction

The global COVID-19 pandemic has affected approximately 620 million individuals and resulted in over 6.5 million deaths [1]. While many patients experience mild or asymptomatic infections, a significant proportion progress to severe disease, including pneumonia, acute respiratory distress syndrome (ARDS), and multi-organ failure. To mitigate the disease progression and reduce mortality rates, various therapeutic strategies have been explored, including antiviral agents, corticosteroids, and monoclonal antibodies [2].

One promising approach is convalescent plasma (CP) therapy, a method of passive immunization that has been employed as a treatment strategy in previous viral outbreaks, such as the influenza pandemic, SARS, MERS, and Ebola. CP contains antibodies from recovered individuals, which can neutralize the virus and modulate the immune response. Notably, during the Influenza A-H1 N1 outbreak, the administration of CP significantly reduced mortality and viral load without serious adverse effects [3, 4]. Similarly, meta-analyses indicate that early administration of CP after symptom onset can improve survival in severe respiratory infections, reinforcing its potential utility in COVID-19 [5]. Evidence suggests that antibodies produced during severe SARS-CoV-2 infections are over 95% effective at hindering viral replication [6, 7]. The diverse repertoire of antibodies in COVID-19 CP, which target distinct spike protein epitopes, further enhances its therapeutic potential [8–10].

The application of COVID-19 CP, rich in antibodies targeting distinct spike epitopes, emerges as a promising therapeutic intervention against SARS-CoV-2 [10–12]. The entry of SARS-CoV-2 into host cells is mediated by its spike protein, which binds to the angiotensin-converting enzyme 2 (ACE2) receptor [9]. This protein has been a focal point for vaccine and therapeutic antibody development due to its crucial role in viral infectivity. CP therapy may confer benefits through various mechanisms, including the direct neutralization of SARS-CoV-2, enhancement of antibody-dependent cellular cytotoxicity (ADCC), and modulation of excessive inflammation, often a key driver of severe COVID-19 complications [12, 13]. Furthermore, the potential anti-inflammatory, anti-thrombotic, and immunomodulatory effects of CP offer prospects for alleviating health complications associated with COVID-19 [14].

However, the efficacy of CP therapy in COVID-19 remains debated. The US Food and Drug Administration (FDA) granted Emergency Use Authorization (EUA) for CP in August 2020. Yet, clinical trials have produced mixed results. For instance, the RECOVERY trial found no significant benefits from low-titer CP, while a separate study from Argentina reported a 48% reduction in severe respiratory disease among elderly patients receiving high-titer CP shortly after symptom onset [15, 16]. These discrepancies underscore the need for further investigation into factors influencing CP efficacy, such as the timing of administration and the health status of recipients [17, 18].

In the context of limited access to monoclonal antibodies and antiviral agents, CP remains a potential therapeutic option, particularly in resource-limited settings where alternative treatments are unavailable or unaffordable. However, challenges such as donor eligibility, standardization of antibody titers, storage conditions, and regulatory compliance must be addressed to optimize its clinical application. Emerging technologies, including machine learning for donor selection and predictive modeling of antibody efficacy, are being explored to enhance CP therapy outcomes [17–19].

Thus, this study retrospectively examines the clinical outcomes of non-immunized COVID-19 patients treated with CP therapy in 2020 and 2021 at a private hospital in Southern Brazil. By analyzing survival rates and inflammatory markers, this study aims to contribute to the ongoing discourse on CP’s efficacy and its role in COVID-19 management.

## Methods

### Patient data collection and ethical statement

This retrospective unicentric cohort study was conducted on patients diagnosed with COVID-19 who were hospitalized at Maternidade e Cirurgia S/A – Hospital do Rocio, located in Campo Largo, Paraná, Brazil, from July 19, 2020, to February 15, 2021. Electronic medical records were utilized to identify eligible patients for inclusion in the study. The study was approved by the Ethics and Research Committee of the Oswaldo Cruz Foundation (Fiocruz - Brasília), under Certificate of Presentation of Ethical Review: 69237023.0.0000.8027. Informed consent was waived due to the retrospective nature of the study, which employed secondary data.

Inclusion and exclusion criteria were strictly adhered to in this study. Eligible participants included hospitalized adults aged 18 years or older diagnosed with or experiencing an exacerbation of COVID-19, confirmed by RT-qPCR or SARS-CoV-2 antigen test from a respiratory tract sample. Participants were required to have undergone C-reactive protein (CRP) testing both at the onset of hospitalization and at the outcome, within 15 days from the onset of symptoms.

Patients were classified based on the severity of their condition, following institutional criteria. *1) Mild cases:* presence of COVID-19 symptoms without shortness of breath, dyspnea, or abnormal chest images. *2) Moderate cases*: evidence of lower respiratory disease during clinical or imaging assessments, with SpO2 > 94% and lung injury ranging from 20% to 50%. *3) Severe cases* are characterized by SpO2 < 94%, PaO2/FiO2 ratio < 300 mm Hg, respiratory rate > 30 breaths/min, and lung injury exceeding 50%.

Exclusion criteria included individuals demonstrating physical examination findings, laboratory abnormalities, or medical history indicative of conditions that might compromise their safety during the study. Additionally, participants with evidence of critical COVID-19 or a history of anaphylactic reactions to blood component transfusions were excluded from the analysis.

Patients were subsequently stratified into two groups at a ratio of 1:2: one patient who received both plasma treatment and standard care, and two patients who received only standard care. The plasma group consisted of individuals who received approximately 200 mL of plasma per bag, in accordance with institutional transfusion protocols, following comprehensive pre-transfusion assessments. This intervention was administered in conjunction with the institution's standard treatment regimen, which included antivirals, antibiotics, steroids, and oxygen supplementation as clinically indicated. Conversely, the control group received only the standard treatment.

### CP donation

Donor recruitment was conducted through targeted social media campaigns, inviting individuals who met specific eligibility criteria: a confirmed history of COVID-19 infection verified by laboratory testing, at least 30 days post-diagnosis, no prior blood transfusions, absence of mechanical ventilation needs, and male gender.

Recruitment and laboratory testing occurred at the Hematology and Hemotherapy Center of Paraná. Eligibility required a minimum anti-SARS-CoV-2 antibody level of 66.18 U/mL in the collected CP. Donations came from individual donors or were pooled from two to five contributors.

Donors underwent comprehensive evaluations, including physical examinations, liver and kidney function tests, and psychological assessments, to ensure their fitness for donation. These evaluations were essential for safeguarding both donor and recipient health.

**Fig. 1:**
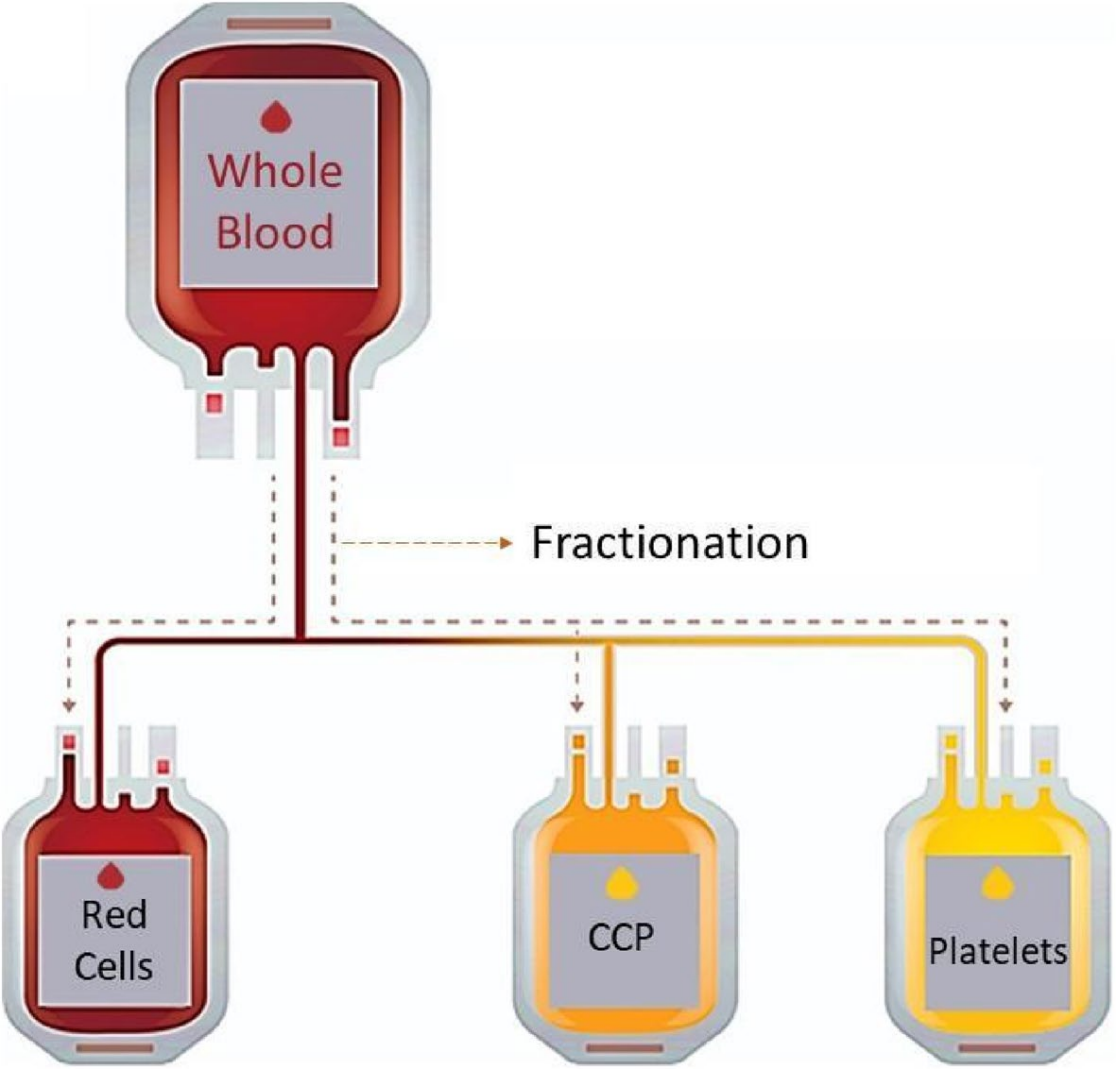
Fractionation process of Convalescent Plasma from whole blood (modified Institute Butantan, 2025)

Collected plasma was processed and tested for HIV, hepatitis B and C, syphilis, HTLV, and Chagas disease, with all batches undergoing stringent quality control before clinical use. Additionally, plasma was categorized by detectable antibody levels against SARS-CoV-2 to enhance therapeutic efficacy. Figure 1 illustrates the fractionation process of CP from whole blood.

### Clinical Information

Clinical data were retrieved from the hospital's electronic medical records system, encompassing various parameters such as demographic characteristics, hospitalization duration from symptom onset, symptomatology, comorbidity profiles, mechanical ventilation requirements, and details of antiviral and steroid therapies administered. Sequential Organ Failure Assessment (SOFA) score (ranging from 0 to 24, with higher scores indicating more severe illness) was recorded. Laboratory data included white blood cell count, lymphocyte count, liver and renal function markers, levels of inflammatory markers, C-reactive protein (CRP), results of thoracic imaging studies, and occurrences of complications such as acute respiratory distress syndrome (ARDS), bacterial pneumonia, and multiple organ dysfunction syndrome (MODS).

### Data elements

Key data elements included: age, BMI, sex, comorbidities (diabetes, hypertension, neoplasia, immunosuppression), pregnancy status, days from symptom onset, C-reactive protein upon admission and discharge, computed tomography (CT) upon admission and discharge, treatments for COVID-19 including CP therapy, ventilatory support (e.g., supplemental oxygen, extracorporeal membrane oxygenation, invasive ventilation, non-invasive ventilation), admission to intensive care unit (ICU), use of vasopressors, hospital mortality, and length of hospital stay. The diagnosis of COVID-19 was confirmed by positive RT-qPCR or rapid antigen test results.

### Statistical analysis

Qualitative variables were expressed using frequency and percentage, while quantitative variables were presented as mean and standard deviation. To compare mean C-reactive protein values on a logarithmic scale between plasma and control groups at patient discharge, an analysis of the covariance (ANCOVA) model was employed. This model considered C-reactive protein measurements at discharge as the dependent variable, treatment type as the independent variable, and C-reactive protein measurements on the first day of treatment as a covariate.

Survival functions for patient follow-up time in days were estimated using Kaplan-Meier analysis for both plasma-treated and control groups, as well as for those categorized by pulmonary involvement based on tomography findings (less than 40% and 40% or more). Comparisons of survival functions were conducted using the log-rank test.

Cox regression models assessed the risk of death from COVID-19 in patients treated with plasma compared to those receiving standard treatment, adjusting for epidemiological and clinical covariates. The dependent variable was the duration from admission to death or discharge, while treatment type (plasma or control) was the independent variable of interest, with covariates including sex, age, comorbidities, and symptom duration. The analysis included both univariate and multivariate stages, retaining covariates with p-values less than 0.05 in the final model. Hazard ratios (HR) and 95% confidence intervals were calculated, with a significance level set at p < 0.05. All statistical analyses were performed using SAS 9.4 software.

## Results

During the observational period (19 July 2020 to 15 February 2021), 350 patient records underwent eligibility screening, resulting in 245 patients meeting inclusion criteria. Of these, 100 patients received CP alongside standard institutional treatment (Plasma Group), and 145 received standard treatment alone (Control Group).

The Control Group had a mean age of 60 (±17.4) years, compared to 55 (±15.2) years in the Plasma Group. Sex distribution was comparable: 55.2% (80/145) of the Control Group and 55% (55/100) of the Plasma Group were female. The Control Group exhibited a mean hospitalization duration of 9.0 days (±10.3) and symptom onset-to-hospitalization interval of 7.4 days (±3.4), while the Plasma Group had a longer hospitalization duration of 11.1 days (±21.2) but a shorter symptom-to-hospitalization interval of 5.5 days (±1.7).

Regarding comorbidities, 38.2% (47) of the Control Group had none, while 61.8% (76) had at least one. Conversely, in the Plasma Group, 43% (43) had no comorbidities, and 57% (57) had at least one. Pulmonary involvement, categorized as 20% to 40%, accounted for 39.3% (33) of cases in the Control Group and 33.9% (19) in the Plasma Group. The epidemiological and clinical characteristics of the patients are presented in Table 1.

**Table 1:** Epidemiological and clinical characteristics of the patients

Variables*	**Group**		*p***-value#**
	**Control**	**Plasma**	
**Sex**			0.73
Female	80 (55.2)	53 (53.0)	
Male	65 (44.8)	47 (47.0)	
**Age**	60 ± 17.4	55 ± 15.2	0.02
**Days of Symptoms**	7.4 ± 3.4	5.5 ± 1.6	< 0.0001
**Days of Hospitalization**	9.0 ± 10.3	11.1 ± 21.2	0.84
**Comorbidities**			0.46
Absent	47 (38.2)	43 (43.0)	
Present	76 (61.8)	57 (57.0)	
**Pulmonary Involvement**			0.22
< 20 %	13 (15.5)	17 (30.4)	
20 % a 40 %	33 (39.3)	19 (33.9)	
40 % a 50 %	17 (20.2)	7 (12.5)	
50 % a 70 %	15 (17.9)	11 (19.6)	
> 70 %	6 (7.1)	2 (3.6)	
**Denouement**			0.06
Discharge	120 (82.8)	91 (91.0)	
Death	25 (17.2)	9 (9.0)	

^*^Values expressed as frequency (%) or mean ± standard deviation

#*p*-value calculated by Chi-square test or Mann-Whitney test

No statistically significant association was observed between the type of treatment (control or plasma) and sex, outcome, comorbidity, and pulmonary involvement (p = 0.7373, p = 0.0667, p = 0.4685, and p = 0.2216, respectively). Similarly, the mean number of hospitalization days did not show a significant difference between the two types of treatment (p = 0.8404). However, the mean age of patients undergoing plasma treatment was significantly lower than that of patients in the control group (p = 0.0189). Additionally, the mean number of symptom days in patients treated with plasma was significantly lower than in the control group (p < 0.0001). For the mean values of C-reactive protein on the logarithmic scale, recorded at discharge and properly adjusted by the initial C-reactive protein values at the beginning of treatment, there was a lack of significant difference between patients treated with plasma compared to those undergoing standard treatment (p = 0.1106). This result suggests a similarity in the impact of plasma and conventional therapy on the C-reactive protein level throughout the treatment period, indicating that both groups showed comparable evolution regarding this important clinical measure (refer to Table 2 for detailed C-reactive protein values).

**Table 2:** C-reactive protein values (logarithmic scale) after treatment using control or plasma

	**Treatment – Mean [95% CI]**		**Comparisons Between Treatments**		
**Variable**	**Control (n = 145)**	**Plasma (n = 100)**	**Difference Control** **versus Plasma [IC 95%]**	**F-value**	p-value*
**C-reactive protein (logarithmic scale)**					
**1 st day of treatment**	4.1 [3.9; 4.3]	3.7 [3.4; 3.9]			
**At the time of discharge**	3.0 [2.8; 3.3]	2.5 [2.2; 2.8]			
^**At the time of discharge (adjusted)#**^	2.9 [2.7; 3.1]	2.7 [2.4; 2.9]	0.3 [−0.06; 0.6]	2.6	0.11

* The p-values for treatment comparisons were computed using analysis of covariance (ANCOVA), with the values from the first day of treatment serving as the covariate

#Means adjusted by the baseline values of the ANCOVA model

**Fig. 2:**
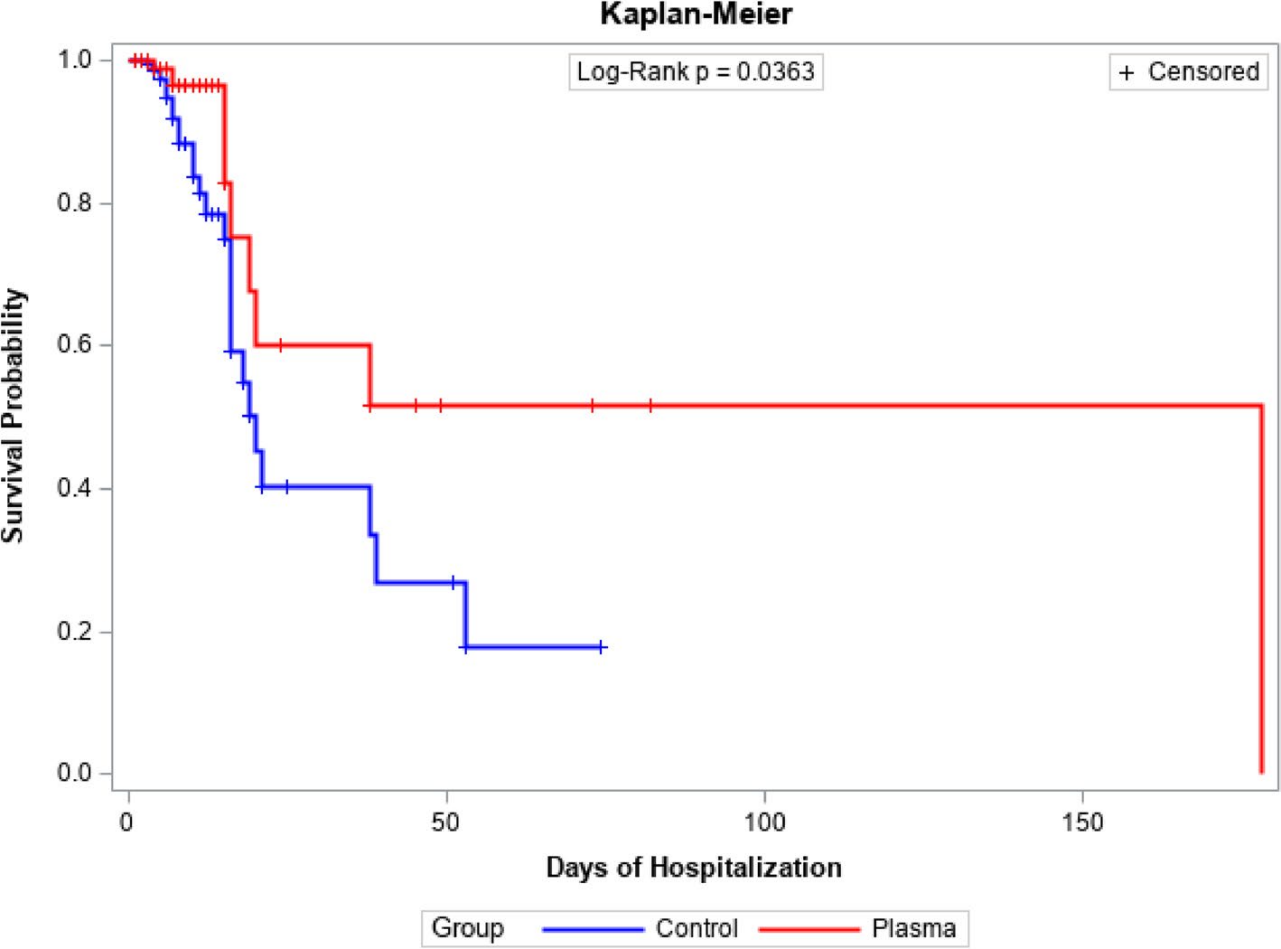
Survival analysis regarding the efficacy of plasma treatment compared to standard treatment during the hospitalization period

In survival analysis assessing the efficacy of plasma treatment versus standard therapy, statistically significant advantages favouring plasma-treated patients are observed (p = 0.0363). This superiority endures beyond immediate comparisons, exhibiting a sustained and noteworthy divergence in survival probability between the cohorts throughout the follow-up period. Figure 2 illustrates the Survival Analysis delineating the efficacy of plasma treatment versus standard therapy during hospitalization.

**Fig. 3:**
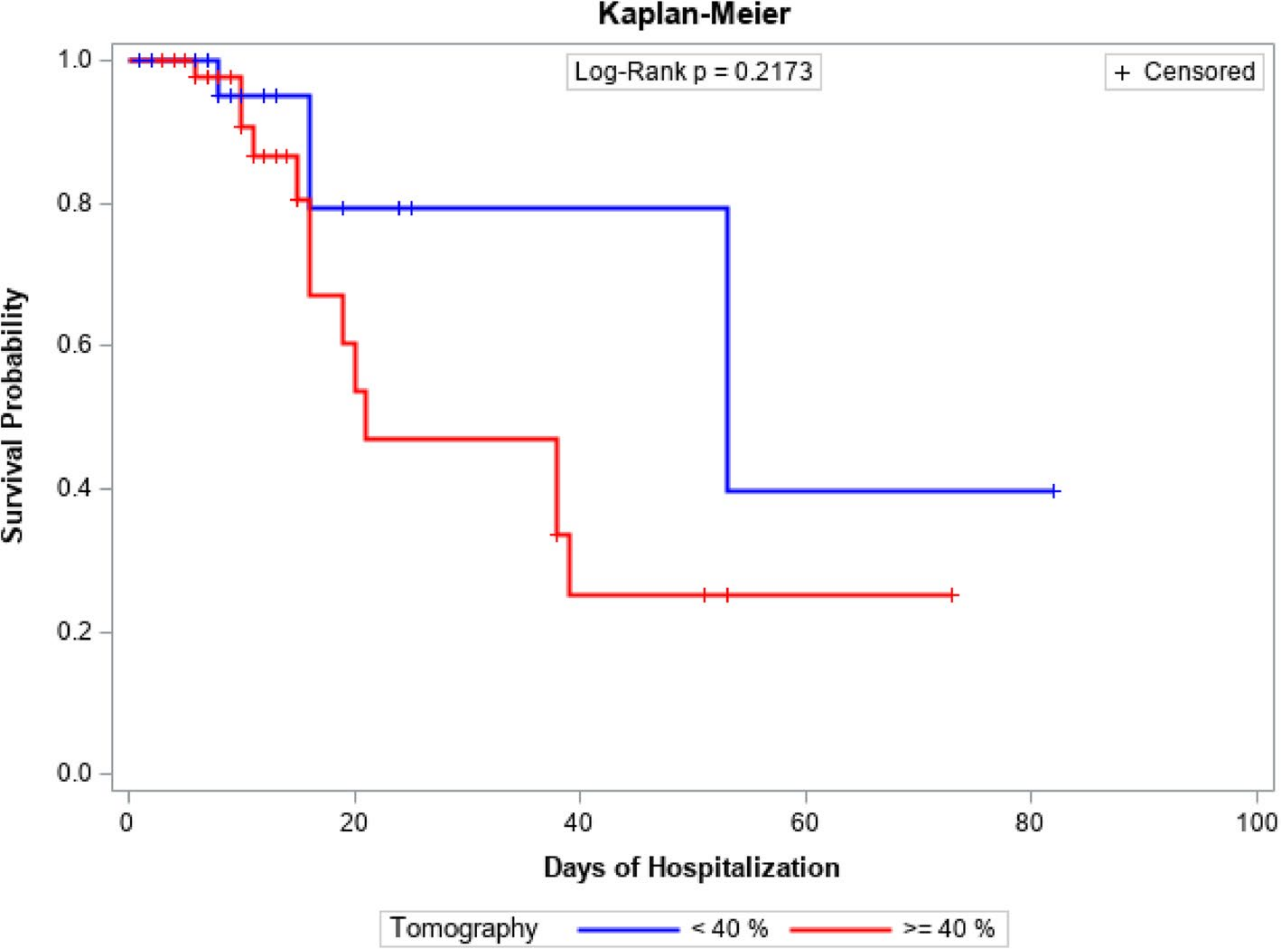
Survival analysis in relation to pulmonary involvement during the hospitalization period

When assessing survival in relation to pulmonary impairment, the data analysis suggests that there is no statistically significant difference in survival probability between patients with pulmonary impairment equal to or exceeding 40% and those with pulmonary impairment below 40%. With a p-value of 0.2173, this indicates that the degree of pulmonary impairment does not serve as a decisive factor in patient longevity during the follow-up period. Hence, the extent of pulmonary impairment does not exert a significant influence on patient survival over time. Figure 3 illustrates the Survival Analysis on pulmonary impairment during hospitalization.

Initially, in the bivariate analysis, only the age variable exhibited a statistically significant p-value (< 0.05), prompting its inclusion in the subsequent multivariate model. Following adjustment for age, the independent variable of interest, treatment, demonstrated statistical significance (p = 0.0480). In the bivariate analysis, treatment displayed a significant association with the time until death (p = 0.0460) when not adjusting for covariates, indicating a 2.26-fold higher risk of death for control group patients compared to those receiving plasma. This association persisted after age adjustment (p = 0.0480), maintaining a nearly constant risk ratio of 2.25 relative to the bivariate analysis. Age, as a covariate, manifested a significant association with time until death in both bivariate and multivariate analyses. In the multivariate model, patients aged 60 years or older exhibited a 4.10-fold higher risk of death compared to those under 60 years old (p = 0.0086). Table 3 presents the distribution of study variables and corresponding hazard ratios, along with 95% confidence intervals, as per the Cox regression model for mortality events (n = 245).

**Table 3:** Distribution of study variables according to crude and adjusted hazard ratios and their respective 95% confidence intervals, as per the Cox regression model for the event of death (N = 245)

	**Gross HR**		HR Adjusted*	
Variables*	**HR (95 % CI)**	***p*-value**	**HR (95% CI)**	***p*-value**
**Sex**		0.97	-	-
Male	1	-	-	-
Male	1.01 (0.50; 2.05)	0.97	-	-
**Age**		0.01		0.01
< 60 years old	1	-	1	-
≥ 60 years old	4.15 (1.44; 11.93)	0.01	4.10 (1.43; 11.74)	0.01
**Symptom Days**		0.57	-	-
≤ 7 days	1	-	-	-
> 7 days	1.24 (0.59; 2.62)	0.57	-	-
**Comorbidities**		0.34	-	-
Yes	1	-	-	-
No	1.51 (0.65; 3.54)	0.34	-	-
**Treatment**		0.04		0.04
Plasma	1	0.04	1	-
Control	2.26 (1.01; 5.02)	0.04	2.25 (1.00; 5.04)	0.04

^*^Adjusted for age and treatment

## Discussion

This retrospective unicenter cohort study aimed to rigorously evaluate the efficacy of CP therapy in conjunction with standard institutional care among COVID-19 patients from 19 July 2020 to 15 February 2021. Beyond immediate treatment outcomes, the study investigated potential long-term effects and prognostic indicators associated with plasma therapy. By analyzing a comprehensive dataset covering various demographic and clinical parameters, including age, gender distribution, baseline comorbidities, and disease severity metrics, the study provides a nuanced understanding of how patient characteristics interact with treatment responses.

Our analysis revealed significant demographic differences between treatment cohorts, with age emerging as a notable determinant. Additional demographic variables such as gender, duration of symptom onset, and hospitalization were considered potential confounders. Detailed clinical assessments provided insights into the patient population's baseline characteristics, including comorbidities and disease severity indices. This thorough characterization was essential for untangling the complex factors affecting treatment outcomes and ensuring the validity of subsequent analyses.

Previous studies have highlighted that demographic factors like male gender, advanced age, and hospitalization are associated with increased antibody responses, suggesting their potential impact on the effectiveness of CP therapy [20]. Our findings align with this, demonstrating significant population heterogeneity across treatment groups and highlighting the need for further investigation into underlying determinants and effect modifiers. Advanced statistical methodologies, such as propensity score matching and subgroup analyses, were employed to mitigate potential biases and enhance the study's reliability. Subgroup analyses stratified by disease severity, symptom duration, and treatment regimen variations offered valuable insights into treatment response variability and informed the development of targeted therapeutic strategies.

CP therapy may have promising potential in improving clinical outcomes and possibly reducing mortality, influenced by factors such as the timing of treatment initiation. This contrasts with previous studies that did not demonstrate a mortality benefit from CP therapy [21–23]. Costa’s study highlights that the primary aim of CP therapy is to provide temporary passive immunity to the recipient, aiding in the control of an active infection. However, a key principle underlying antibody-based treatments is the importance of timing. Antibody preparations are most effective when administered prophylactically or at the early stages of disease progression [14]. Differences in study designs, timing of plasma administration, and participant age might account for these discrepancies. The variation in evidence across studies reflects the disease's complexity and highlights the need for continued research to fully uncover CP therapy's benefits and refine its application [24–26].

Salazar et al found CP therapy to be safe and effective, with 76% of patients showing a one-point improvement in clinical status on the WHO scale. Our study identified substantial population heterogeneity among treatment groups, which requires further investigation into underlying determinants and effect modifiers. Despite discrepancies in age and symptom duration, statistical associations with other factors such as sex, comorbidities, and pulmonary involvement were not significant [27].

A randomized, double-blind, controlled trial of CP in adults with severe COVID-19 showed results where the CP was not associated with a significant improvement in clinical status but led to a significant improvement in mortality in hospitalized adults with severe COVID-19 [25]. This finding is supported by recent research, which suggests that CP can be an effective treatment, especially when administered early and with high antibody titers [28].

The findings of Bohoněk et al, a retrospective observational study conducted in the Czech Republic with 1,498 patients, of whom 406 received convalescent plasma (CP), indicate a significant association between CP treatment and improved survival rates (79% vs. 62% in the control group). Notably, the greatest benefits were observed when CP was administered within the first three days following symptom onset. In contrast, our unicentric retrospective cohort study compared 100 patients treated with CP to 145 who received standard treatment. While we also observed an enhancement in survival among those receiving CP, our analysis revealed no statistically significant differences in C-reactive protein (CRP) levels. This suggests that the benefits of CP may be more closely linked to immunomodulatory effects rather than a direct impact on viral load. While both studies support the potential advantages of CP therapy, they also emphasize the critical importance of timing in administration, whereas our findings prompt further exploration into the immunological mechanisms at play [29].

However, some studies have reported contrasting results. For instance, one study found that CP did not reduce the risk of intubation or death at 30 days and was associated with more severe adverse events [7]. A comprehensive meta-analysis reinforces these findings, suggesting that CP is not associated with lower all-cause mortality or better disease progression, regardless of initial disease severity or baseline antibody levels [30]. Additionally, comparisons between CP and standard care have shown no significant differences in 28-day mortality or other relevant clinical outcomes [31].

Despite these mixed results, a recent literature review by Kandula et al emphasizes the potential benefits of CP when used appropriately. The review also highlights that high-titer CP and agrees with our findings, when administered early in the course of illness, has been associated with reduced risk of hospitalization and mortality, particularly in patients who are unable to receive other antiviral treatments. Furthermore, CP has been historically effective in treating other respiratory infections like SARS, H1 N1, and MERS, suggesting that it can play a valuable role in managing severe viral infections [28].

CP has generally been well-tolerated, but there's limited information on serious adverse events, as demonstrated in Hoffmann's study, CP was well tolerated. Similarly, other studies have reported no significant safety concerns when high-titer CP is administered to patients with COVID-19 [32]. To fully assess treatment effects, secondary endpoints like hospitalization duration, mechanical ventilation needs, and thrombotic events were carefully evaluated. Exploratory analyses also looked at biomarkers, such as inflammatory cytokine profiles and viral load dynamics, to understand the immunological mechanisms influenced by CP therapy. It was observed that when administered early with high antibody titers, CP can reduce hospitalization time, the need for mechanical ventilation, and thrombotic events.

Biochemical monitoring is crucial for assessing disease severity and progression in patients affected by SARS-CoV-2. Studies have shown contrasting information, for instance Chen et al. suggested that the risk of developing severe events increases by 5% for each unit increase in C-reactive protein concentration in COVID-19 patients. In contrast, our study, which analyzed the behavior of average CRP values at admission and outcome, identified no significant differences between treatments. However, it is important to consider that C-reactive protein levels can be influenced by a range of pre-existing conditions in patients, which may introduce variability in the results [33].

Analysis of clinical endpoints, including survival and time to mortality, yielded significant insights into the prognostic implications of CP. Longitudinal assessments of patient-reported outcomes, quality of life measures, and post-treatment functional status provided a holistic perspective on treatment efficacy and patient well-being. Survival analysis evaluating the efficacy of plasma versus standard therapy showed statistically significant advantages favouring patients treated with plasma. This superiority persisted beyond immediate comparisons, showing a sustained and notable divergence in survival probability between cohorts throughout the follow-up period.

Multivariate analysis identified age and CP therapy as significant determinants of time to mortality, highlighting their respective roles in shaping treatment outcomes. The application of CP in COVID-19 patients with pneumonia has shown a correlation with more favourable outcomes. Advanced age, ICU admission, diabetes, and pre-existing cardiovascular conditions were identified as independent predictors of mortality within 28 days [27].

The aging process is correlated with significant and comprehensive changes in the body's response to COVID-19, and specific immunological alterations are likely to play a crucial role in increased mortality among elderly patients [34]. Age over 70 years, smoking, obesity (BMI over 30), and diabetes are identified as significant independent predictors of 30-day mortality among hospitalized patients with severe COVID-19 pneumonia treated with CP infusion [35]. Exploratory analyses investigating potential gene-expression signatures predictive of treatment response were undertaken to unravel novel molecular biomarkers indicative of treatment efficacy, paving the way for personalized therapeutic approaches tailored to individual patient profiles.

Demographic factors such as sex, age, and hospitalization status are correlated with antibody responses in CP therapy for COVID-19. Transcriptomic analyses have revealed significant changes in gene expression that could serve as biomarkers for disease progression and treatment response. Additionally, exosomes derived from CP present a new therapeutic pathway with potential for immune modulation and precision diagnostics. These findings collectively contribute to the understanding of molecular biomarkers for COVID-19 treatment efficacy and pave the way for personalized therapeutic approaches [36–38].

Some studies reveal a lack of consistency in survival prediction, with neutralization titers not reversing the decline in fatal cases. On the other hand, an association is observed between higher antibody affinity and sustained elevated IgA responses with better survival outcomes [38]. Furthermore, transcriptomic analyses indicate that COVID-19 patients exhibit altered gene expression related to antiviral defence, immune response, and risk of secondary infections. However, CP and corticosteroid treatments have been associated with a reduction in these blood expression signatures, suggesting potential benefits in modulating the immune response and mitigating secondary complications [40].

Studies on cell-free plasma cell RNA (cfRNA) profiles reveal positive regulation of antiviral genes and genes related to neutrophil activation in COVID-19 patients. CP treatment appears to lead to a decrease in the expression levels of these genes, suggesting a dynamic inflammatory response that can be modulated by therapy [39, 40].

These findings suggest that adjunctive CP therapy may confer a significant survival benefit in COVID-19 patients, particularly among younger cohorts. Nonetheless, further investigation is warranted to validate these findings and elucidate the underlying mechanisms governing treatment responses comprehensively. Future research should prioritize refining patient selection criteria, optimizing treatment protocols, and elucidating the immunological mechanisms modulated by CP therapy to maximize treatment efficacy and improve patient outcomes.

In conclusion, our study reinforces the findings that CP serves as an effective therapeutic option for improving survival among non-immunized COVID-19 patients. The retrospective analysis indicated that patients receiving CP alongside standard treatment had a significantly higher survival rate (91%) compared to the control group (82.8%), particularly when administered early and to those with moderate disease severity. While the study highlights critical determinants such as age and the potential of predictive biomarkers, it also notes the variability in treatment responses across different demographic and clinical groups. Overall, CP emerges as a promising solution, given its availability, safety profile, and low cost, especially in settings with limited access to advanced treatments. Future research should focus on elucidating the mechanisms of action of CP and optimizing its integration into broader therapeutic strategies.

## Data Availability

No datasets were generated or analysed during the current study.
